# Burnout level and associated factors in a sub-Saharan African medical setting: prospective cross-sectional survey

**DOI:** 10.1186/s12909-020-02194-2

**Published:** 2020-09-10

**Authors:** Jean-Rodolphe Mackanga, Emeline Gracia Mouendou Mouloungui, Josaphat Iba-ba, Pierre Pottier, Jean-Baptiste Moussavou Kombila, Jean-Bruno Boguikouma

**Affiliations:** 1 Center for Training and Research in Pedagogy of Health Sciences - Faculty of Medicine, Strasbourg, 4 rue Kirschlager, F-67085 Strasbourg, France; 2 Department of Internal Medicine and Medical Specialties, University of Health Sciences, Libreville, post box 4009, Libreville, Gabon; 3 Department of Internal Medicine, University of Nantes, 1 rue Gaston Veil - post box 53508, 44035 Nantes Cedex1, France

**Keywords:** Burnout, Factors, Gabon, Medical setting, Prevalence

## Abstract

**Background:**

Burnout in the hospital environment is a problem that affects care and training. Often explored in the high-income medical context, burnout is poorly studied in low and middle-income countries characterized by a precarious hospital situation and a high stake linked to the Millennium Development Goals. The aim of our study was to determine in medical practitioners, in a sub-Saharan African country’s medical context, the burnout level and associated factors.

**Methods:**

A prospective cross-sectional study by using a self-administered Likert-scale questionnaire addressed to doctors and doctoral medical students in Gabon. Maslach Burnout Inventory scale has been used. Burnout symptoms were defined by high level in at least one of the 3 dimensions. Severe burnout defined by high level in all dimensions. Explored factors: socio-demographic and psychometric. Multiple logistic regression has been performed.

**Results:**

Among 104 participants, severe burnout prevailed at 1.9% (95% CI: 0.2–6.8%) and burnout symptoms at 34.6% (95% CI: 25, 6–44.6%). The associated factors with burnout symptoms: age (OR = 0.86, *p* = 0.004), clinical activity in a university hospital center (OR = 5.19, *p* = 0.006), the easy access to the hospital (OR = 0.59, *p* = 0.012), number of elderly dependents living with the practitioner (OR = 0.54, *p =* 0.012), place of residence (same borough where the hospital is located: OR = 4.09, *p* = 0.039) and to be favorable to traditional medicine (OR = 1.82, *p* = 0.087). Nagelkerke’s R-squared:53.1%.

**Conclusion:**

In Gabon, middle-income country, almost one practitioner in two has burnout symptoms. The young age, the university hospital center, the difficulty to access to hospital and to live in the borough where the hospital is located increase the probability of burnout symptoms. These results must put question to relevant authorities regarding health and medical education, to set up: a public transport for practitioners, an optimal primary health care system, a regulation of medical tasks in hospitals, a training in clinical supervision.

## Background

The hospital is an environment where medical practice and medical training take place simultaneously. In health systems, the well-being of hospital practitioners is essential for good quality of care [1], and to achieve public health objectives [2]. Thus, a discomfort in hospital environment have a negative effect on the quality of care and medical education. Among the uncomfortable phenomena affecting hospital practitioners as physicians or medical students, burn-out is widely cited. Several definitions of burnout are described in the literature, but the description by Maslach and Jackson is the most used, defining burnout by three components or symptoms: emotional exhaustion, depersonalization and the reduction of personal accomplishment [3]. Burnout is quite studied in the social and medical context of developed countries [4], and this has allowed many socio-legal advances in favor of workers. Moreover, in line with these advances, recently in May 2019, the World Medical Association (WMA) welcomed the decision of the World Health Assembly to classify burnout syndrome as having an impact on health status and to incorporate it into the new version of the International Classification of Diseases of the World Health Organization (WHO) [5]. The WMA, through the voice of its president, urged that this decision of the WHO lead very soon to the adoption of a new approach, which will take into account multiple factors, including the working conditions of doctors across the world. However, in low- and middle-income countries that are characterized by modest quality of health care, medium medical training level and poor hospital conditions, burnout is still insufficiently studied.

Thus, the aim of the study was to determine in medical doctors and doctoral students, in the medical context of a middle-income sub-Saharan African country, the burnout level and the associated factors, in order to hypothesize potential etiological factors of burnout. This would guide research about cause and effect relationship in order to define specific actions, which could improve: the well-being of practitioners, the quality of care, and the medical training in these precarious conditions.

## Methods

### Study design

According to the aim of our study, a prospective cross-sectional survey was conducted.

#### Inclusion criteria and study settings

The study concerned consenting medical practitioners, regardless of age or gender. These included medical doctors and doctoral medical students working part-time in clinical practice in Gabon at the moment of the study. Gabon is a sub-Saharan African middle-income country. It is populated by about 1.8 million inhabitants with about 3 to 4 physicians per 10,000 inhabitants [6], or approximately a total between 540 to 720 physicians. But these physicians are unequally distributed on the national territory [7].

#### Sample size

We determined the minimum sample size required for our survey based on an acceptable margin error of 5%, a confidence level of 95% and an expected prevalence of severe burnout of approximately 7%, average prevalence reported in the literature [8]. Thus, by applying the formula *n =* z^2^ x p (1 - p) / m^2^ [9] (z: for 95% = 1.96, p: assumed prevalence = 7% and m: acceptable margin error = 5%), a minimum of 100 practitioners were required.

#### Sampling and data collection

**Fig. 1:**
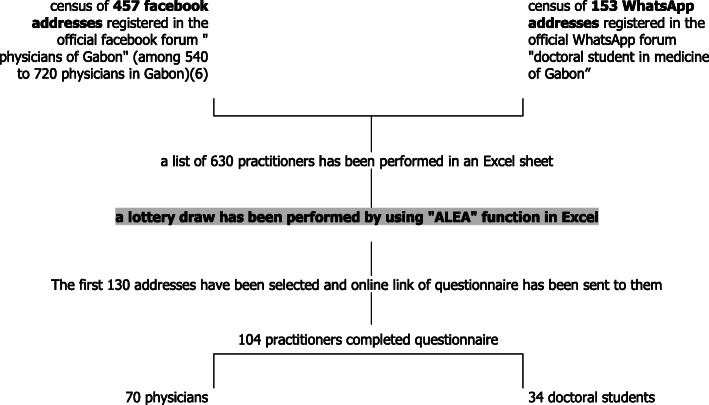
Sampling of study population

The source population was physicians and medical doctoral students working in clinical practice in Gabon and registered on the professional Facebook forum of physicians in Gabon, and the WhatsApp forum of doctoral medical students in Gabon. The Facebook forum of physicians is a professional online forum (named “physicians of Gabon”) where are registered 457 physicians on a total of 540 to 720 physicians in Gabon [6]. They represent around 63.5 to 84.6% of physicians in Gabon according to medical density and population size. All doctoral medical students are registered in a WhatsApp forum. In fact, in Gabon, there is only one medical school, and doctoral medical students were 153 in Gabon at the moment of study. For academic needs, all doctoral students are registered in a WhatsApp forum to facilitate communication in medical school of Gabon. These online forums bring together the majority of medical practitioners and all medical doctoral students in Gabon. Thus, we estimated that these forums had representative population of physicians and medical doctoral students for the study. We made a list on Excel sheet 2016 using Facebook addresses of the 457 physicians and WhatsApp addresses of the 153 doctoral medical students. The Facebook addresses are easily accessible to each physician registered in the professional Facebook forum, and the WhatsApp addresses were available from the student delegate. Probability sampling was performed using the *ALEA* function in Excel 2016. According to sample size expected, the first 100 (+ 30%) addresses were selected and the link of anonymous questionnaire (with a cover text that succinctly explained the rationale of the study) has been sent to these online addresses. From time to time, reminders were necessary to mobilize the participants. The practitioners who answered the anonymous questionnaire according to their consent, were our study population (Fig. 1). Each anonymous questionnaire completed and submitted online was systematically collected on the database via google form. Data collection took place from November 1, 2018 to April 1, 2019. Data exported in Excel sheet to statistical analysis software.

#### Variables operationalization

- Practitioners feelings about burnout were collected by using the validated French version of Maslach Burnout Inventory - Human Services Survey for Medical Personnel (MBI-HSS (MP)), widely used [10]. Therefore, the following outcomes criteria have been defined:

• severe burnout defined by high level score in all 3 dimensions of the Maslach Burnout Inventory Scale: emotional exhaustion (EE) ≥27 and depersonalization (DP) ≥10 and personal achievement (PA) ≤33. Severe burnout was coded as a binary categorical variable (yes = 1, no = 0).

• burnout (or burnout symptom) defined by the existence of at least one high level score in one of the 3 dimensions of the Maslach Burnout Inventory Scale: EE score ≥ 27 or DP score ≥ 10 or AP score ≤ 33. The burnout (the burnout symptom) was coded in binary categorical variable (yes = 1, no = 0).

- the psychometric data related to the perception of the practitioners were collected by a self-administered Likert-scale questionnaire at 7 levels (proportionally to assertion worded) and treated as a numeric variable. These were the following parameters: *a) the easy access to the hospital*: in Gabon, public transport is poor and this suggests that the difficulty of access to the workplace could affect motivation, and therefore indirectly interacts to burnout. Thus, we have identified it as a potential factor to explore. *b) the weekly frequency of the professional medical activity outside the public hospital:* organization and equipment in private hospitals would make the working environment more satisfactory than in public hospitals. Or, working in the two hospitals could be overworked. Thus, we have identified it as a potential factor to explore. *c) the frequency of activity in non-professional associations*: taking part in extra-professional associative activities could be an environment that promotes humanization, or empathy skill, which is inversely correlated to burnout. Or, working simultaneously in non-professional associations could be overworked. Thus, we have selected it as a potential factor to explore. *d) opinion favorable to traditional medicine*: traditional medicine has a preponderant psychological orientation, supposing an understanding listening skill, which could interact with depersonalization feeling. Also, traditional medicine could be an alternative medicine where practitioners suffering of burnout symptoms would refer patients. Thus, we selected “to be favorable to traditional medicine” as potential factor to explore.

- demographic and socio-professional data: gender, marital status, hospital status (doctor versus doctoral medical student), hospital facility attended (university hospital center (UHC) versus other), extra-professional associative activity (yes versus no), means of transport (taxi versus personal car), place of residence relative to hospital (to live in same borough versus to live in different borough where the hospital is located) were coded as dichotomous categorical variables. Age, number of dependent children (living with the practitioner), number of elderly dependents (living with the practitioner), estimated average number of patients taken care of per day were coded as numerical variables.

### Statistical analysis

#### Descriptive study

Categorical variables were summarized as a percentage expressed with a 95% confidence interval (95% CI). The none normality of distributions of majority of numeric variables has been checked by One-Sample Kolmogorov-Smirnov test. Thus, numeric variables have been summarized by median with interquartile range (IQR).

#### Analytical study

- univariate analysis to evaluate the link between each variable and burnout symptoms was performed by Fisher’s Chi-square test for categorical variables and the Mann-Whitney U-test for numeric variables. Variables associated to burnout symptoms with statistical significance less than 25% (*p* < 0.25) were selected for the multivariate analysis. In view of the exploratory nature of our study which tested original factors, we chose 25% to reduce the risk of excluding possible factors masked by confounding factors [11]. Age, sex and place of residence relative to hospital (same or different borough from that of the hospital) were considered as forced variables for multivariate analysis.

- multivariate analysis was performed by binary multiple logistic regression using backward stepwise method (with Wald’s statistic). The adjusted odd ratio (aOR) was the estimator of the association between the burnout symptoms event and potential factor. Backward stepwise method generated a final model of probable predictive factors of prevalent burnout symptoms. The thresholds for the probability of entering and removing factor in the model were set at 5 and 10% respectively. The quality of the final model of potential predictor factors was evaluated by the Nagelkerke’s R-squared and the Hosmer-Lemeshow test. The French version of the SPSS 21 software (Statistical Package for the Social Sciences) was used.

## Results

### Characteristics of the study population

One hundred and four (104) medical practitioners completed the questionnaire. Majority groups were: female gender (sex ratio male (M) / female (F) = 0.52 (95% CI: 0.35–0.79), doctors (versus doctoral students) 67.3% (95% CI 57.4–76.2%), performing extra-professional associative activity 75.0% (95% CI: 65.6–83.0%) (Table 1).

**Table 1:** Characteristics of the study population (*N* = 104 (100%)

Age (year)	Mean, IQR		35.0	30.3–40.0
Gender				
female	n, %, CI 95%.	68	65.4%	55.4–74.5%
male	n, %, CI 95%.	36	34.6%	25.6–44.6%
Matrimonial status				
single	n, %, CI 95%.	42	40.4%	30.9–50.5%
living maritally	n, %, CI 95%.	62	59.6%	49.5–69.1%
Place of residence and hospital				
different borough	n, %, CI 95%.	56	53.9%	43.8–63.7%
same borough	n, %, CI 95%.	48	46.2%	36.3–56.2%
Means of transport				
taxi	n, %, CI 95%.	52	50.0%	40.0–60.0%
personal car	n, %, CI 95%.	52	50.0%	40.0–60.0%
Easy access to the hospital (self-assessment / scale from 0 to 7)	Median, IQR		4.0	3.0–5.0
Hospital				
Other	n, %, CI 95%.	42	40.4%	30.9–50.5%
University Hospital Center (UHC)	n, %, CI 95%.	62	59.6%	49.5–69.1%
Hospital status				
doctoral student in medicine	n, %, CI 95%.	34	32.7%	23.8–42.6%
Doctor (physician)	n, %, CI 95%.	70	67.3%	57.4–76.2%
Extra-professional associative activity				
yes	n, %, CI 95%.	78	75.0%	65.6–83.0%
no	n, %, CI 95%.	26	25.0%	17.0–34.5%
Estimated number of patients supported per day	Median, IQR		10.0	10.0–17.3
Number of elderly dependents living with the practitioner at home	Median, IQR		0.0	0.0–1.0
Number of dependent children living with practitioner at home	Median, IQR		2.0	1.0–3.0
Attendance of extra-professional associations (self-assessment / scale from 0 to 7)	Median, IQR		2.0	1.0–4.0
Frequency of medical activity outside public hospital per week (self-assessment / scale from 0 to 7)	Median, IQR		2.0	1.0–3.8
To be favorable to traditional medicine (self-assessment / scale from 0 to 7)	Median, IQR		1.0	1.0–2.0
Emotional Exhaustion (from 0 to 54)	Median, IQR		18.0	10.0–26.8
Depersonalization (from 0 to 30)	Median, IQR		8.0	3.0–11.8
Personal Achievement (from 0 to 48)	Median, IQR		40.5	36.0–42.8

### Burnout prevalence

The prevalence of severe burnout was at 1.9% (95% CI 0.2–6.8%). The burnout symptoms prevalence was at 34.6% (95% CI: 25.6–44.6%): ie EE ≥27: 17.3% (95% CI: 10.6–26.0%), DP ≥10: 25.0% (95% CI: 17.0–34.5%) and PA ≤33: 7.7% (95% CI: 3.4–14.6%) (Table 2).

**Table 2:** Prevalence of burnout in Gabonese medical settings. (Total *N* = 104)

	n	%	CI 95%	
**Burnout symptom (Emotional Exhaustion: EE)**				
High emotional exhaustion	18	17.3%	10.6%	26.0%
Moderate emotional exhaustion	38	36.5%	27.3%	46.6%
Low emotional exhaustion	48	46.2%	36.3%	56.2%
**Burnout symptom (Depersonalization: DP)**				
High depersonalization	26	25.0%	17.0%	34.5%
Moderate depersonalization	34	32.7%	23.8%	42.6%
Low depersonalization	44	42.3%	32.7%	52.4%
**Burnout symptom (Personal Accomplishment: PA)**				
Low Personal accomplishment	8	7.7%	3.4%	14.6%
Moderate Personal accomplishment	40	38.4%	29.1%	48.5%
High Personal accomplishment	56	53.9%	43.8%	63.7%
**Burnout symptoms: EE ≥ 27 or DP ≥ 10 or PA ≤ 33**	36	34.6%	25.6%	44.6%
**Severe burnout: EE ≥ 27 and DP ≥ 10 and PA ≤ 33**	2	1.9%	0.2%	6.8%

### Associated factors to burnout symptoms

Associated factors of burnout symptoms in univariate analysis: younger age (*p* < 0.0001), low number of dependent children (*p* = 0.044), low score regarding “easy access to the hospital” (*p* = 0.012), low score regarding “medical activity outside public hospital” (*p <* 0.0001), single (OR = 2.61 95% CI: 1.14–6.00); medical student status (OR = 3.25 95% CI: 1.37–7.69) and activity in University Hospital Center (UHC) OR = 3.50 95% CI: 1.40–8.77 (Tables 3 and 4).

**Table 3:** Link between numeric variables and burnout symptoms in Gabonese medical settings (bivariate analysis)

analyzed factors	Burnout symptoms				p
	Yes (*N* = 36)		No (*N* = 68)		
	Median	IQR	Median	IQR	
Age (year)	30	29–34	38.5	32–41	< 0.0001
Number of elderly dependents living with the practitioner at home	0	0–0	0	0–1	0.203
Number of dependent children living with practitioner at home	1.5	1–2	2	1–3	0.044
Estimated number of patients supported per day	15	10–20	10	10–15	0.188
Easy access to the hospital (scale 0 to 7)	3	1–5	4	4–5	0.012
Attendance of extra-professional associations (scale from 0 to 7)	2	1–4	2	1–4	0.281
Frequency of medical activity outside public hospital per week (scale 0 to 7)	1	1–2	2	1–4	< 0.0001
To be favorable to traditional medicine (scale 0 to 7)	1.5	1–2	1	1–2	0.142

**Table 4:** Link between categorical variables and burnout symptoms in Gabonese medical settings (bivariate analysis)

Variables	modality	Burnout symptoms		Total = 104	OR	CI 95%		p
		Yes (=36)	No (=68)					
Gender	female	24	44	68	1.09	0.47	2.56	1
	male	12	24	36	reference	1		
Matrimonial status	single	20	22	42	2.61	1.14	600	0.035
	living maritally	16	46	62	reference	1		
Means of transport	taxi	22	30	52	1.99	0.87	4.54	0.149
	Personal car	14	38	52	reference	1		
Hospital	UHC	28	34	62	3.5	1.4	8.77	0.007
	Other	8	34	42	reference	1		
Extra-professional associative activity	no	12	14	26	1.93	0.78	4.79	0.163
	yes	24	54	78	reference	1		
Hospital status	Doctoral student	18	16	34	3.25	1.37	7.69	0.008
	Doctor (physician)	18	52	70	reference	1		
Place of residence and hospital	different borough	20	36	56	1.11	0.49	2.5	0.83
	Same borough	16	32	48	reference	1		

### Model of predictors of burnout symptoms (multivariate analysis)

After logistic regression and adjusted, the following factors were retained in the predictive model: age in years (aOR = 0.86 95% CI: 0.78–0.96; 0.004), activity in UHC (aOR = 5.19 95% CI: 1.61–16.75, *p* = 0.006), score regarding “ easy access to the hospital” (aOR = 0.59 95% CI: 0.40–0.89, *p* = 0.012), the number of elderly dependents living with the practitioner (aOR = 0.54 95% CI: 0.33–0.88, *p =* 0.012), to live in different borough (versus same borough) where the hospital is located (aOR = 0.24 95% CI: 0.06–0.93, *p* = 0.039) and the favorable opinion score for traditional medicine (aOR = 1.82 95% CI: 0.92–3.61, *p* = 0.087). The statistics of Hosmer-Lemeshow = 18.7% and Nagelkerke’s R-squared 53.1% (Table 5).

**Table 5:** Model of burnout symptoms predictors in Gabonese medical settings (multivariate analysis)

Factors	Coefficients	aOR^a^	CI 95%		p	statistics of model	
						Nagelkerke’s R-squared	Hosmer-Lemeshow’s test
University Hospital Center (versus other hospital)	1.647	5.19	1.61	16.75	0.006	53.1	0.187
To be favorable to traditional medicine (self-assessment/scale 0 to 7)	0.598	1.82	0.92	3.61	0.087		
Age (year)	−0.15	0.86	0.78	0.96	0.004		
Easy access to the hospital (scale from 0 to 7)	−0.521	0,.59	0.40	0.89	0.012		
Number of elderly dependents living with the practitioner at home	−0.616	0.54	0.33	0.88	0.012		
Place of residence: same (vs different) borough from than of the hospital	−1.409	4.09	1.07	15.59	0.039		

^a^ adjusted odd ratio (aOR), with burnout symptoms as dependent variable

## Discussion

### Burnout prevalence

The high prevalence of burnout symptoms reflects work-related suffering in addition to the very difficult working conditions initially described in public hospitals in Gabon [12]. Given the prevalence of burnout symptoms in our study, it can be said that in the Gabonese medical settings, nearly half of all medical care situations are at risk of an unsatisfactory clinical outcome. Indeed, it is established that the existence of burnout symptoms is associated with an unfavorable clinical outcome for patients as opposed to an empathy that has an opposite effect [13–15]. Moreover, the data of our study show that the most prevalent symptom of burnout in Gabon’s medical community is depersonalization (DP), and it is well established that this dimension of burnout means a low level of empathy [14]. This observation contributes more to explain the feeling of discomfort in the caregiver-patient relationship often reported by patients attending public hospitals [12] and forebodes the poor professional temperament in public hospitals [15]. However, the prevalence of burnout symptoms in Gabon’s medical community is close to the values ​​reported in the French, American (United States of America) and Asian medical settings [8, 14, 16–18].

### Factors associated with burnout symptoms in Gabonese medical settings

Young age is strongly associated with burnout symptom in Gabonese medical settings. With each increase of 1 year of age, the probability of having a burnout symptom decreases by 14%. This result could be explained by a difficult phase of professional acculturation. A period where the young practitioner adapts to the work environment. Also, the young practitioner is often victim of the phenomenon of sliding tasks. Indeed, it is not uncommon to note the absence of a transparent regulation on the delegation of administrative or medical tasks entrusted to young practitioners or medical students. And, we can also evoke the probably overly directive relationship between the supervisor practitioner and the young supervised practitioner, while this relationship should be more collaborative and more cooperative. It is not uncommon for medical students to be treated more prominently as poorly listened employees and this, at the expense of their learner status. Indeed, medical student status as resident is also widely recognized as a factor associated with burnout symptoms in most studies [8].

The low score regarding “easy access to the hospital” is strongly associated with burnout symptoms. An improvement of one point on the Likert scale from 0 to 7, decreases the probability of burnout symptoms by 40%. This result could be explained by difficulties in accessing the hospital due to the lack of public transport, the poor condition of urban roads, which enclaves large urban areas [19]. The urban enclaved areas are sources of pre-hospital demotivation. This demotivation contaminates the feeling at work and expose to burnout. These pre-hospital demotivation factors, can also explain the low practitioners performance [15]. Also, as our results show that young age is associated to burnout symptoms, it is more probable that students and young physicians who have modest income face more transport difficulties to get to hospital.

Working in the University Hospital Center (UHC) multiplies by 5 the probability of burnout symptoms. This could be explained by the frequent lack of care materials in UHC where the largest number of patients converge. This situation forces practitioners to devote a large part of their activity to solving social problems than medical problems. This context lowers the feeling of personal accomplishment, increases emotional exhaustion, and promotes depersonalization as a defense mechanism against the psychic pain of being unable to effectively solve patients’ problems [20].

Living in the borough where the hospital is established is paradoxically associated with burnout. This result questions about inconveniences to be availability due to the proximity to workplace. This exposes the young practitioner to work overtime and be solicited during holidays. Also, living near the hospital can mean a modest economic conditions (students and young doctors), that motivate young practitioner for doing medical activity additionally and exposes them to burnout. The evaluation of the geographical distribution of the residences of the practitioners in relation to the hospitals according to the socio-economic level will need to be explored.

For each dependent elderly person living with the practitioner, the probability of having burnout symptoms decreases by 46%. This suggest that a practitioner caring for the elderly at home improves cognitive empathy. And empathy is inversely correlated with burnout.

Finally, the trend towards the association between the opinion score favorable to traditional medicine and the symptoms of burnout questions the interaction between burnout and the perception of certain social representations concerning norms, values ​​and the beliefs of practitioners. Also, for practitioners, alternative medicine like traditional medicine could be a solution against burnout symptoms. This result needs qualitative study for more exploration, in order to understand how the caregiver-patient relationship operates in the acculturation of caregiver regarding his medical belief.

### Possible solutions to reduce the symptoms of burnout in the Gabonese medical context

Hospital is the only place where young doctors and students acquire their medical skills. But, the well-being (in the learning environment) which is a determining factor for learning seems seriously impaired in Gabonese hospitals. In view of the above (university hospital center, low score regarding “easy access to the hospital” and young age as predictive factors of burnout symptoms), optimal medical learning and best health care are uncertain in this medical context. For this, some points must put question to relevant authorities regarding health and medical education, namely: 1-the construction of social housing near hospitals for resident or young practitioners, 2-set up a public transport system, 3-to promote a regulation of medical tasks in hospitals, 4- to promote optimal system of primary health care for helping UHC and 5- set up a training in clinical supervision for senior doctors. This could reduce the symptoms of burn-out in young practitioners, improve the well-being of practitioner in their medical practice and promote medical learning.

The burnout topic is relevant, justifying our study which aimed to characterize the epidemiology of burnout in a precarious hospital environment in a sub-Saharan African medical setting. The survey design of the study and the statistical analysis are adequate to the research question. The statistical model of factors associated with burnout symptoms could explain burnout symptoms event at over 53% according to Nagelkerke’s R-squared adjusted (Table 5). This result indicates the potential etiological factors that will need to be explored in appropriate study design to verify a cause and effect relationship. Regarding to the physicians number (less than a thousand) in Gabon at the time of the study and the probability sampling performed, our study population is potentially representative [6, 7]. However, the sampling has been performed by using facebook addresses obtained online through official facebook forum of physicians of Gabon and lacked stratification (two populations studied: doctoral students and doctors) could be a limit of our study. But, ratio between both populations in our study was in the expected range according to the source population, which minimize bias.

## Conclusion

In medical settings of middle-income Gabon, almost one practitioner in two has burnout symptoms with a predominance of depersonalization. The young age, the University Hospital Center, to live in same borough where the hospital is located, the difficulty of access to the hospital and the lower number of elderly dependent adult living with the practitioner increase the probability of burnout symptoms. These results must put question to relevant authorities regarding health and medical education, namely: social housing of young practitioners, public transport, regulation of medical tasks, set up optimal system of primary health care for helping the university hospital center and promoting training in clinical supervision. The link between burnout symptoms and the opinion score favorable to traditional medicine suggest the need to understand how the doctor-patient relationship operates in the acculturation process of medical practitioners in Gabon.

## Data Availability

The datasets used and/or analyzed during the current study are available from the corresponding author on reasonable request.

## Abbreviations

CEI: Institutional Ethics Committee
CERMEL: Medical Research Center of Lambaréné
CI: Confidence Interval
DP: Depersonalization
EE: Emotional Exhaustion emotional
F: Female
IQR: Inter Quartile Range
M: Male
OR: Odd ratio
p: *p*-value
PA: Personal accomplishment
SPSS: Statistical Package for the Social Sciences
UHC: University Hospital Center
vs: Versus
WHO: World Health Organization
WMA: World Medical Association
